# Antifungal Susceptibility Profiles and Resistance Mechanisms of Clinical *Diutina catenulata* Isolates With High MIC Values

**DOI:** 10.3389/fcimb.2021.739496

**Published:** 2021-10-29

**Authors:** Xin-Fei Chen, Wei Zhang, Xin Fan, Xin Hou, Xiao-Yu Liu, Jing-Jing Huang, Wei Kang, Ge Zhang, Han Zhang, Wen-Hang Yang, Ying-Xing Li, Jin-Wen Wang, Da-Wen Guo, Zi-Yong Sun, Zhong-Ju Chen, Ling-Gui Zou, Xue-Fei Du, Yu-Hong Pan, Bin Li, Hong He, Ying-Chun Xu

**Affiliations:** ^1^ Department of Laboratory Medicine, State Key Laboratory of Complex Severe and Rare Diseases, Peking Union Medical College Hospital, Chinese Academy of Medical Science and Peking Union Medical College, Beijing, China; ^2^ Graduate School, Chinese Academy of Medical Science and Peking Union Medical College, Beijing, China; ^3^ Department of Laboratory Medicine, Beijing Key Laboratory for Mechanisms Research and Precision Diagnosis of Invasive Fungal Diseases (BZ0447), Beijing, China; ^4^ Clinical Microbiology Laboratory, The First Affiliated Hospital of Hebei North University, Zhangjiakou, China; ^5^ Department of Infectious Diseases and Clinical Microbiology, Beijing Chaoyang Hospital, Beijing, China; ^6^ Department of Medical Research Center, Peking Union Medical College Hospital, Chinese Academy of Medical Science & Peking Union Medical College, Beijing, China; ^7^ Department of Laboratory Medicine, Daqing Oilfield General Hospital, Daqing, China; ^8^ Department of Laboratory Medicine, The First Affiliated Hospital of Harbin Medical University, Harbin, China; ^9^ Department of Laboratory Medicine, Tongji Hospital, Tongji Medical College, Huazhong University of Science and Technology, Wuhan, China; ^10^ Department of Laboratory Medicine, The Fourth Affiliated Hospital of Harbin Medical University, Harbin, China; ^11^ Department of Clinical Laboratory, Fujian Medical University Union Hospital, Fuzhou, China; ^12^ Department of Clinical Laboratory, The Affiliated Hospital of Qingdao University, Qingdao, China

**Keywords:** *Diutina catenulata* (*Candida catenulata*), antifungal susceptibility, *ERG11*, *FKS1*, gene mutation, drug resistance mechanisms

## Abstract

*Diutina catenulata* (*Candida catenulata*) is an ascomycete yeast species widely used in environmental and industrial research and capable of causing infections in humans and animals. At present, there are only a few studies on *D. catenulata*, and further research is required for its more in-depth characterization and analysis. Eleven strains of *D. catenulata* collected from China Hospital Invasive Fungal Surveillance Net (CHIF-NET) and the CHIF-NET North China Program were identified using matrix-assisted laser desorption ionization–time of flight mass spectrometry and internal transcribed spacer sequencing. The antifungal susceptibility of the *Diutina catenulata* strains was tested using the Clinical and Laboratory Standards Institute broth microdilution method and Sensititre YeastOne™. Furthermore, ERG11 and FKS1 were sequenced to determine any mutations related to azole and echinocandin resistance in *D. catenulata*. All isolates exhibited low minimum inhibitory concentration (MIC) values for itraconazole (0.06–0.12 μg/ml), posaconazole (0.06–0.12 μg/ml), amphotericin B (0.25–1 μg/ml), and 5-flucytosine (range, <0.06–0.12 μg/ml), whereas four isolates showed high MICs (≥4 μg/ml) for echinocandins. Strains with high MIC values for azoles showed common *ERG11* mutations, namely, F126L/K143R. In addition, L139R mutations may be linked to high MICs of fluconazole. Two amino acid alterations reported to correspond to high MIC values of echinocandin, namely, F621I (F641) and S625L (S645), were found in the hot spot 1 region of *FKS1*. In addition, one new amino acid alteration, I1348S (I1368), was found outside of the *FKS1* hot spot 2 region, and its contribution to echinocandin resistance requires future investigation. *Diutina catenulata* mainly infects patients with a weak immune system, and the high MIC values for various antifungals exhibited by these isolates may represent a challenge to clinical treatment.

## Introduction

In recent years, *Candida* infections have been on the rise worldwide (Sanguinetti et al., 2015; Pristov and Ghannoum, 2019). Although *Candida albicans* remains the main causative agent of these infections, the rate of non–*C. albicans* candidial infections is increasing (Sanguinetti et al., 2015). Candida infections are not usually multidrug-resistant, but there are notable cases of drug resistance among non-albicans Candida (NAC) species. *Candida auris* shows *in vitro* multidrug resistance and is associated with outbreaks. *Candida glabrata* is the most common cause of candidemia and is resistant to azoles and echinocandins. *Candida parapsilosis* is another notorious pathogen isolated from patients, which causes outbreaks and multidrug resistance (Arastehfar et al., 2021). These *Candida* species, though rare, are clinically common and necessitate a better understanding of their pathogenic mechanisms. *Diutina catenulata* (*C. catenulata*), an ascomycete, can colonize the digestive tract of animals and humans and cause superficial or deep infections (Crozier, 1977; Radosavljevic et al., 1999; Ha et al., 2018; Cafarchia et al., 2019). *Diutina catenulata* is usually utilized in the production of industrial products (Joo et al., 2008; Subramanya et al., 2017; Babaei and Habibi, 2018; Cafarchia et al., 2019). *Diutina catenulata* belongs to the CTG-Ser clade and is closely related to *Saccharomyces cerevisiae* (O'Brien et al., 2018). The first time describing *D. catenulata* associated with disease in humans in 1977, and *D. catenulata* was recovered from the nail of a 50-year-old male Australian patient (Crozier, 1977). Subsequently, the first case of candidemia was diagnosed in a patient with cancer (Radosavljevic et al., 1999).

There are few reports of human infection with *D. catenulata*, and only a small number of studies are available to guide clinical treatment. *Diutina catenulata* generally exhibits antifungal sensitivity (Radosavljevic et al., 1999; Ha et al., 2018); however, some strains showing resistance to azoles and echinadines have been isolated from eggs and feces (Glushakova et al., 2021). Thus, it is important to consider the potential antifungal resistance of *D. catenulata*. However, the resistance mechanism of *D. catenulata* is poorly understood. Amino acid alternations in Erg11p and Fks1p are responsible for causing azole and echinocandin resistance in pathogenic *Candida* spp. (Berkow and Lockhart, 2017; Toutounji et al., 2019). However, the complete open reading frames of both *ERG11* and *FKS1* genes have not been revealed, and the gene polymorphisms of *ERG11* and *FKS1* in clinical *D. catenulata* isolates remain unknown.

Despite the three cases reported worldwide, the occurrence of *D. catenulata* in clinical specimens of the Chinese population remains unelucidated. Thus, we studied the antifungal susceptibility and potential drug resistance mechanisms of the clinical isolates of *D. catenulata* obtained in China over a period of 9 years.

## Material and Methods

### Ethics Statement

This study was approved by the Human Research Ethics Committee of Peking Union Medical College Hospital (No. S-263). A written informed consent was obtained from all study participants to examine the isolates cultured from them for scientific research.

### Isolates

During the period from 2010 to 2018 (**Table 1**), 11 *D. catenulata* isolates were collected from seven different hospitals in five provinces from the China Hospital Invasive Fungal Surveillance Net (CHIF-NET) and the CHIF-NET North China Program. These isolates were mainly from invasive fungal infection specimens. Most specimens were obtained from patients with bloodstream infections, which indicates the seriousness of the situation for these patients.

**Table 1:** List of isolates included in the study.

Strain	Gender/Age	Year	Department	Source of isolate	Identity	Clinical diagnosis
1	F/75	2010	ICU	Blood	100	Pneumonia
2	M/56	2010	ICU	Blood	100	Coronary heart disease, lung infection
3	M/54	2013	Organ transplantation	Ascites	100	Primary liver cancer
4	M/60	2016	Neurosurgery	Cerebrospinal fluid	100	Hydrocephalus
6	M/79	2016	ICU	Blood	100	Coronary heart disease
11	M/2	2015	Hematopathy	Blood	100	Infectious fever
12	F/59	2018	ICU	Blood	100	Cerebral hemorrhage
13	M/45	2017	Organ transplantation	Ascites	100	Liver transplantation
15	M/84	2016	ICU	Urine	100	Subarachnoid hemorrhage
16	M/81	2017	ICU	Urine	100	Fever
17	F/78	2017	ICU	Urine	100	Closed abdominal trauma

### Identification

All isolates were identified at the species level using matrix-assisted laser desorption ionization–time of flight mass spectrometry (MALDI-TOF MS) conducted with Autof MS 1000 (Autobio, Zhengzhou, China) and Vitek MS (Bio Merieux, Marcy-l’Étoile, France). The species identification was confirmed *via* the sequencing of the rDNA internal transcribed spacer (ITS) region (ABI 3730XL, Thermo Fisher Scientific, Cleveland, OH, USA). PCR and sequencing of the amplicons were performed using the forward primers, V9G and ITS1 (5′-TCCGTAGGTGAACCTGCGG-3′), and the reverse primers, LS266 and ITS4 (5′-TCCTCCGCTTATTGATATGC-3′) (Zhang et al., 2014; Hou et al., 2016). The phylogenetic tree of *D. catenulata* was constructed by alignment with the ITS gene sequences of the common *Candida* species in the NCBI gene library. Maximum-likelihood phylogenetic trees were constructed with IQ-TREE using an ultrafast bootstrap approximation approach with 1,000 replicates (Trifinopoulos et al., 2016).

### Antifungal Susceptibility Testing

Minimum inhibitory concentrations (MICs, μg/ml) of nine antifungal agents, namely, anidulafungin, micafungin, caspofungin, fluconazole, posaconazole, voriconazole, itraconazole, amphotericin B, fluorocytosine, were determined for all isolates using the Sensititre YeastOne™ system (SYO, Trek Diagnostic Systems, Thermo Fisher Scientific, Cleveland, OH, USA) according to the manufacturer’s instructions. MICs of micafungin (Astellas, Japan), caspofungin (CAS; Merck, USA), fluconazole [(National Institute for Food and Drug Control (NIFDC), China], voriconazole (NIFDC, China), amphotericin B (NIFDC, China), and flucytosine (NIFDC, China) were determined using the CLSI broth microdilution method. MICs were determined after 36 h of culture. *Candida krusei* ATCC 6258 and *C. parapsilosis* ATCC 22019 were used as quality control strains. When the MIC obtained by the two methods fell within a twofold dilution gradient, the essential agreement (EA) between Sensititre™ YeastOne™ and CLSI was considered for *D. catenulata.* The clinical breakpoints of antifungals against *D. catenulata in vitro* have not yet been established by the Clinical and Laboratory Standards Institute (CLSI) or the European Committee on Antimicrobial Susceptibility Testing (EUCAST). Consequently, the interpretative criteria were followed to test the *in vitro* susceptibility of *Candida* spp. according to the CLSI M59 guidelines (CLSI, 2017).

### DNA Extraction and Sequencing of *ERG11* and *FKS1*

On comparing published amino acid sequences of *ERG11* (3641571, 1466526) and *FKS1* (3639844, 856398) with *D. catenulata* CBS 565 whole-genome sequences (PJEZ00000000.1) and conducting gene annotation, we identified the *ERG11* and *FKS1* homologous proteins in *C. catenulata.* Amino acid alignment of Erg11p and Fks1p from published data and whole-genome sequencing of analyzed data is shown in **Supplementary Figures 1**, **2**.

### *ERG11* and *FKS1* Sequencing

Genomic *ERG11* and *FKS1* were amplified by PCR using specific primers and sequenced as previously described. The forward (5′- ACATTATTTATTGCCCCATG-3′) and reverse primers (5′-GCAAGTATCCCGCTTTTCCC-3′) for *ERG11* and those for *FKS1* are shown in **Supplementary Table 1**.

### Nucleotide Sequence Accession Numbers

The ITS region sequences of strains found in this study were deposited in GenBank with accession numbers MW624477 to MW624482.

### Literature Review

This literature review considered the available data regarding the susceptibility of the *D. catenulata* species to antifungals. The literature search was performed on September 3, 2021, using the following three databases: PubMed (https://pubmed.ncbi.nlm.nih.gov), Web of Science (https://webofknowledge.com), and Embase (https://www.embase.com). The terms “*Diutina catenulates*” or “*Candida catenulate*” or “*Candida ravautii*” were entered in the category of “Title/Abstract” in the PubMed Advanced Search Builder, and “TS=(*Diutina catenulata*)” or “TS=(*Candida catenulata*)” or “TS=(*Candida ravautii*)” was entered in the Web of Science databases. The search in Embase was conducted in the advanced search area, including the terms “‘diutina catenulate’:ti,ab,kw” or “‘candida catenulata’:ti,ab,kw” or “‘candida ravautii’:ti,ab,kw.” Studies describing the patients infected with *D. catenulata* were selected and summarized.

## Results

### Species Identification of *D. catenulata* Using MALDI-TOF and DNA Sequencing

**Figure 1:**
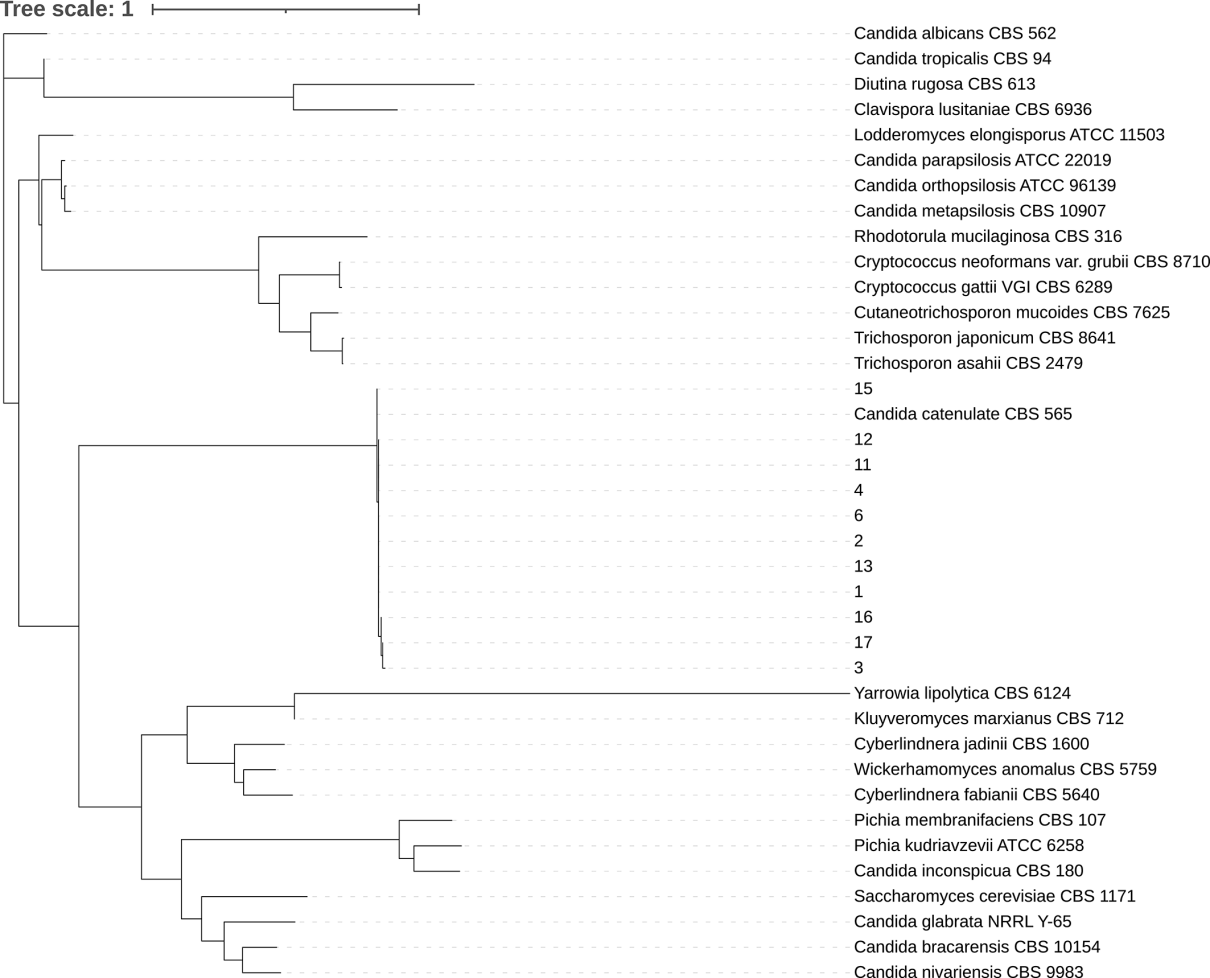
Phylogenetic tree. Internal transcribed spacer (ITS) sequences of nuclear rDNA of *Candida* spp. were used. The ITS sequence of the model strain was obtained from NCBI gene bank. CLC sequence viewer software was used for sequence alignment and IQ-Tree ultrafast bootstrap with 1,000 replicates was used for tree construction.

All 11 clinical isolates were identified as *D. catenulata* by the Autof MS 1000 and Vitek MS. The ITS sequences of the study isolates exhibited 100% sequence identity to the corresponding ITS sequences from the reference *D. catenulata* isolates in GenBank (*C. catenulata* CBS 565). We performed a phylogenetic analysis using ITS sequences (**Figure 1**).

This study comprised 72.7% (8/11) males and 27.3% (3/11) females, with an average age of 58 years. Among the specimens, blood accounted for 45.5% (5/11), urine for 27.3% (3/11), ascites for 18.2% (2/11), and cerebrospinal fluid for 9.1% (1/11). The patients belonged to the following departments: intensive care unit (ICU) 45.5% (5/11), organ transplant department 18.2% (2/11), neurosurgery department 18.2% (2/11), hematology department 9.1% (1/11), and healthcare department 9.1% (1/11).

### Antifungal Susceptibility

The quality control strains (*C. krusei* ATCC 6258 and *C. parapsilosis* ATCC 22019) showed MICs within the expected ranges. Aggregated MIC distributions of the nine antifungal agents tested against *D. catenulata* isolates by YeastOne™ are shown in **Table 2**. For all 11 *D. catenulata* isolates, the MICs of itraconazole and posaconazole were in the range of 0.06 to 0.12 μg/ml. In addition, 63.6% (7/11) of *D. catenulata* isolates exhibited high MIC values for fluconazole (MIC >8 μg/ml) and 81.8% (9/11) showed high MIC values (MIC ≥4 μg/ml) for caspofungin. A total of 54.5% (6/11) of *D. catenulata* isolates showed MICs of ≥1 μg/ml for anidulafungin. A total of 36.4% (4/11) of *D. catenulata* isolates showed high MICs for micafungin (MIC ≥1 μg/ml). The MICs of amphotericin B and 5-flucytosine against all *D. catenulata* isolates ranged from 0.25 to 1 and <0.06 to 0.12 μg/ml, respectively. Four *D. catenulata* isolates exhibiting increased MICs of ≥1 μg/ml for all three echinocandins were obtained in our study. Furthermore, the MIC values of echinocandins and fluconazole were high for five isolates of *D. catenulata*.

**Table 2:** Epidemiological cutoff values (ECV) of nine antifungal agents based on aggregated minimum inhibitory concentration (MIC) distributions of *Diutina catenulata*.

	MIC (μg/ml)											Range	Mode*^a^*	ECV (μg/ml)*^b^* at:		
	<0.06	0.06	0.12	0.25	0.5	1	2	4	8	>8				95%	97.50%	99%
Fluconazole								4		7		4–256	4	256	256	256
Voriconazole		1	3	2	2	2		1				0.12–4	0.12	4	4	4
Itraconazole		5	6									0.06–0.12	0.12	0.12	0.12	0.12
Posaconazole	1	6	4									0.06–0.12	0.06	0.12	0.12	0.12
Caspofungin		1	1		1			2		6		0.06–>8	>8	>8	>8	>8
Micafungin	4		2		1				1	3		0.03–>8	<0.06	>8	>8	>8
Anidulafungin		2	2		1	2		2		2		0.06–>8	0.06, 0.12, 1, 4, >8	>8	>8	>8
5-Flucytosine	10		1									<0.06–0.12	<0.06	0.12	0.12	0.12
Amphotericin B				1	5	5						0.25–1	1	1	1	1

^a^Most frequent MIC.

^b^Calculated ECVs comprising ≥95, ≥97.5, or ≥99% of the statistically modeled MIC population.

### Agreement Between the CLSI Method and Sensititre YeastOne™

The EA values of the MICs between the CLSI method and YeastOne™ for a majority of the antifungal drugs tested were >90%. In triazoles, 100% EA values were obtained for amphotericin B and 5-flucytosine. EA values for caspofungin and micafungin were 81.9 (9/11) and 91% (10/11), respectively (**Supplementary Table 2**).

### Sequence Analysis of the *ERG11* Gene in the Clinical Isolates

The complete open reading frame of *D. catenulata ERG11* gene, predicted using bioinformatics analysis, showed that *ERG11* comprises 1,563 base pairs (**Table 3**). The *ERG11* gene was sequenced for all 11 *D. catenulata* isolates. These isolates displayed amino acid substitutions in Erg11p compared with the sequences in GenBank. The SNP F126L (C378G) and synonymous mutations C681T, C888A, C999T, and C1164T were found in two isolates with high fluconazole MICs (>32 μg/ml). Amino acid substitutions, F126L (C378G) and G417K (L139F), and synonymous mutations, C681T, C888A, C999T, and C1164T, were found in three isolates with fluconazole MICs >64 μg/ml. Additionally, one isolate with K143R (A428G) and synonymous mutations, C681T, C888A, C999T, and C1164T, showed high resistance to fluconazole (MIC >256 μg/ml). Only one resistant strain had the K143R (A428G) mutation. In addition, synonymous mutations G306A and C852T were found in three isolates (**Table 4**). By aligning the amino acid sequences of Erg11P from *C. albicans* and *S. cerevisiae*, we found that Erg11p of *D. catenulata* shared 69.75 and 65.25% identity with that of *C. albicans* and *S. cerevisiae*, respectively. Notably, the *C. catenulata* Erg11p contained residues at three locations (F126, L139, K143) that corresponded with three residues (F126, L139, K143, respectively) in *C. albicans* ERG11 (**Supplementary Figure 1**) whose alterations (F126L, K143R) have been reported to be associated with azole resistance (Berkow and Lockhart, 2017).

**Table 3:** The variability of *ERG11* and *FKS1* genes in clinical *Diutina catenulata* isolates.

	DNA sequence			Protein sequence	
	Length (nt)	No. of SNPs	No. of allele types	Amino acid changes	No. of protein types
ERG11	1563	10	5	3	3
FKS1	5652	27	9	7	7

**Table 4:** The variability of *ERG11* gene in clinical *D. catenulata* isolates.

Isolate	Missense mutation	Synonymous mutation				MICs (μg/ml)			
	Nucleotide mutation (amino acid mutation)					FZ*^a^*	VOR*^a^*	PZ*^a^*	IZ*^a^*
1	A428G (K143R)	C681T	C888A	C999T	C1164T	256	0.5	0.12	0.12
2	C378G (F126L)	C681T	C888A	C999T	C1164T	64	1	0.12	0.12
3		G306A	C852T			4	0.12	0.06	0.12
4		G306A	C852T			4	0.12	0.06	0.06
6	C378G (F126L)	C681T	C888A	C999T	C1164T	32	0.25	0.06	0.06
11						4	0.12	0.06	0.06
12	A428G (K143R)					64	0.25	0.12	0.12
13		G306A	C852T			4	0.06	0.03	0.06
15	C378G (F126L), G417K (L139F)	C681T	C888A	C999T	C1164T	256	4	0.12	0.12
16	C378G (F126L), G417K (L139F)	C681T	C888A	C999T	C1164T	128	1	0.06	0.12
17	C378G (F126L), G417K (L139F)	C681T	C888A	C999T	C1164T	64	0.5	0.06	0.06

^a^FZ, Fluconazole; VOR, Voriconazole; PZ, Posaconazole; IZ, Itraconazole.

### Sequence Analysis of *FKS1* Gene in Clinical Isolates

The complete open reading frame of *D. catenulata FKS1*, determined by bioinformatics analysis, showed that *FKS1* comprises 5,652 base pairs (**Table 3**). The *FKS1* gene was sequenced for all 11 *D. catenulata* clinical isolates. We found 27 SNPs (A591G, G732C, T1119C, C1194T, G1203A, C1299T, G1365T, A1554G, T1605C, T1861A, C1874T, T2079C, C2100T, C2682T, C2781G, G2871A, G3036A, C3273T, G3367A, T4043G, T4062G, G4230C, C4462T, C4704T, T4977C, C5006T, and 5088C) (**Supplementary Table 3**). The SNP T4062G was found in an isolate with high aggregated MIC distributions for echinocandin (≥8 μg/ml). By aligning the amino acid sequence of Fks1p of *D. catenulata* with those of the corresponding proteins in *C. albicans* and *S. cerevisiae* (which had 83.51 and 71.29% identities with *D. catenulata*, respectively), we found seven amino acid substitutions in the Fks1p of *D. catenulata* (**Supplementary Table 3**). Amino acid substitution F621I was found in one isolate (Isolate 2) with capofungin MIC **>**8 µg/ml. Substitution I1348S was found in three isolates with echinocandin MIC ≥4 µg/ml (isolates 15, 16, and 17). Additionally, amino acid substitutions S625L and F1354L were found in one isolate with an MIC ≥8 μg/ml for three types of echinocandins (Isolate 13) (**Table 5**).

**Table 5:** The variability of *FKS1* gene in the hot spot 1 and near hot spot 2 regions in clinical *D. catenulata* isolates.

Isolate	Missense mutation in *FKS1* hot spot		MICs (μg/ml)					
	Nucleotide mutation (amino acid mutation)		AND*^a^*		MF*^a^*		CAS*^a^*
Hot spot 1								
2		T1861A (F621I), G3367A (G1123S)	1		0.5		>8	
6		T1861A (F621I), G3367A (G1123S)	1		0.12		>8	
13		C1874T (S625L), T4062G (F1354L)	>8		8		>8	
Around hot spot 2								
15		G3367A (G1123S), T4043G (I1348S)	>8		>8		>8	
16		G3367A (G1123S), T4043G (I1348S)	4		>8		>8	
17		G3367A (G1123S), T4043G (I1348S)	4		>8		>8	

^a^AND, Anidulafungin; MF, Micafungin; CAS, Caspofungin.

### Literature Review

Only three of the sourced articles had reported clinical cases of infection with *D. catenulata*, including one case, in which the *D. catenulata* was isolated from the nails and two cases of *D. catenulata* isolated from blood culture (Crozier, 1977; Radosavljevic et al., 1999; Ha et al., 2018). One of the patients was diagnosed with gastric cancer and the other with endocarditis. One of the isolates showed low MICs for amphotericin B, miconazole, econazole, tioconazole, ketoconazole (1 μg/ml), traconazole (0.25 μg/ml), and fluconazole (16 μg/ml). Other isolates showed low MICs for fluconazole and micafungin (2 and 0.06 μg/ml, respectively). In addition, based on the patients’ clinical information, we can conclude that broad-spectrum antibiotic therapy may be a risk factor for infection with *D. catenulata* (**Supplementary Table 4**).

## Discussion

With the development of identification technologies, such as MALDI-TOF MS and high-throughput sequencing, many hitherto unknown species of microorganisms have been precisely identified (Qiu et al., 2019). *Diutina catenulata* is a rare yeast that causes infections in humans (Radosavljevic et al., 1999; Ha et al., 2018; Çakır et al., 2019). Therefore, we determined the proportion of *D. catenulata* isolates among clinical strains recovered from patients using CHIF-NET and the CHIF-NET North China Program. DNA sequencing and MALDI-TOF MS can be used to identify *D. catenulata* at the species level, and the proportions of the different species in the sample can be compared. ITS sequencing identified all 11 isolates, and the consistency of these clinical isolates with CBS 565 was 100%. Currently, ITS sequencing is considered a reliable marker for fungal identification (Wang et al., 2012).

Most *Candida* species are pathogens that cause infections in immunocompromised patients (Cernakova et al., 2019). In this study, *D. catenulata* infection mainly occurred in elderly male admitted ICU patients, and nearly half of the infections were bloodstream infections. There are differences in drug resistance rates among *Candida* species (Sanguinetti et al., 2015). *Candida auris* has attracted much attention worldwide due to its high drug resistance and high fatality rates (Lone and Ahmad, 2019). Despite the low frequency of the species, the high MIC values observed for *D. catenulata* potentially could be important. *In vitro* antifungal susceptibility results showed that all *D. catenulata* showed low MICs for itraconazole, posaconazole, amphotericin B, and 5-flucytosine, but four isolates showed high MICs (≥4 μg/ml) for the echinocandins. More than 36.4% of the isolates showed high MIC values for two echinocandins. High MICs of caspofungin may predict high MICs of micafungin and anidulafungin. More than 72.7% of the isolates showed high MICs for fluconazole. Thus, we suggest that *D. catenulata* with high MICs for fluconazole should be evaluated for its susceptibility to other antifungals. Micafungin has excellent effects *in vivo* against *D. catenulata* in infants (Çakır et al., 2019). However, in the present study, our *in vitro* antifungal susceptibility test showed that 36.4% of isolates showed high MICs (≥4 μg/ml) for echinocandins.

To analyze the molecular mechanisms of the antifungal resistance of *D. catenulata*, we sequenced the *ERG11* and *FKS1* genes. In agreement with the azole drug susceptibility profiles, we found three mutations in *ERG11* of *D. catenulata.* Interestingly, F126L and K143R are the most prevalent residue substitutions in *ERG11* (Berkow et al., 2017), but the L139F substitution is an unreported new observation. The mutations in *FKS1* showed polymorphism. We identified two mutations in *FKS1* hot spot 1 [F621I (F641) and S625L (S645)] and one near *FKS1* hot spot 2 [I1348S (I1368)] associated with high MICs of caspofungin (Toutounji et al., 2019). S625L (S645) has been reported to cause failure of micafungin treatment against *C. albicans* (Slater et al., 2011). G3367A was found in isolates showing high and low MIC values against caspofungin and therefore may not be a potential of high MIC values against this antifungal. Of note, caspofungin was found to have a high interlaboratory variation (Espinel-Ingroff et al., 2013) and therefore the high MIC observed for this antifungal may not necessarily be a cause of concern as long as the MIC of the micafungin and anidulafungin are included and remain low. Polymorphisms of *D. catenulata* may also be used as a basis for typing.

Compared with the three articles have been described, we have noticed that the MICs of fluconazole and echinocandin of some of our strains were higher than 64 and 4 µg/mL, respectively, which are significantly higher than previous studies. This obvious difference also shows that there may be some evolutionary differences between Chinese and foreign isolates. In addition, our ICU patients also are treated with antibiotics. Therefore, patients maybe at high risk of *D. catenulata* infection. Similar to the case with most studies, lack of detailed clinical data on antifungal treatment is a major limitation of our study, which precludes us from reaching a conclusive statement about the choice of antifungal treatment and their potential outcome.

In conclusion, because of the low isolation rates, epidemiological knowledge of rare species is critical for clinical treatment. To the best of our knowledge, this study is the first to report the occurrence and distribution of *D. catenulata* in China, and its findings are expected to guide clinical treatment of *D. catenulata* infection in the future.

## Data Availability Statement

The datasets presented in this study can be found in online repositories. The names of the repository/repositories and accession number(s) can be found in the article/**Supplementary Material**.

## Author Contributions

X-FC and WZ conceived and designed the experiment. XF, WK, GZ, HZ, W-HY, J-WW, D-WG, Z-YS, Z-JC, L-GZ, X-FD, Y-HP, BL, and HH contributed reagents/materials/analysis tools. X-FC, X-YL, J-JH, Y-XL, and WZ performed the experiments. X-FC and WZ analyzed the data and wrote the manuscript. Y-CX, XF, XH, and Y-XL revised the manuscript. All authors contributed to the article and approved the submitted version.

## Funding

This work was supported by the Natural Science Foundation of Beijing, China (Grant No. 7204288), National Natural Science Foundation of China (82002178 and 81971979), Special Foundation for National Science and Technology Basic Research Program of China (2019FY101200), Beijing Municipal Science and Technology Project (Z181100001618015), Beijing Key Clinical Specialty for Laboratory Medicine-Excellent Project (No. ZK201000), and Scientific Research Foundation Project of Hebei Provincial Health Commission (Grant No. 20210702, 20190904, 20180843).

## Conflict of Interest

The authors declare that the research was conducted in the absence of any commercial or financial relationships that could be construed as a potential conflict of interest.

## Publisher’s Note

All claims expressed in this article are solely those of the authors and do not necessarily represent those of their affiliated organizations, or those of the publisher, the editors and the reviewers. Any product that may be evaluated in this article, or claim that may be made by its manufacturer, is not guaranteed or endorsed by the publisher.
