# Supplementary material for: Antifungal Susceptibility Profiles and Resistance Mechanisms of Clinical Diutina catenulata Isolates With High MIC Values
Source: Front Cell Infect Microbiol. 2021 Oct 29;11:739496. doi: 10.3389/fcimb.2021.739496 (PMC8586209; doi:10.3389/fcimb.2021.739496)
Supplement: Supplementary file 1 [file DataSheet_1.docx]

Supplementary Material

# Supplementary Figures and Tables

## Supplementary Figures

**Supplementary Figure 1.** Amino acid sequence alignment of *ERG11* from *Candida albicans*, *Saccharomyces cerevisiae*, and *Candida catenulata*. Three amino acid residues whose alterations had been linked to azole resistance in *C. albicans* are circled with red ovals.


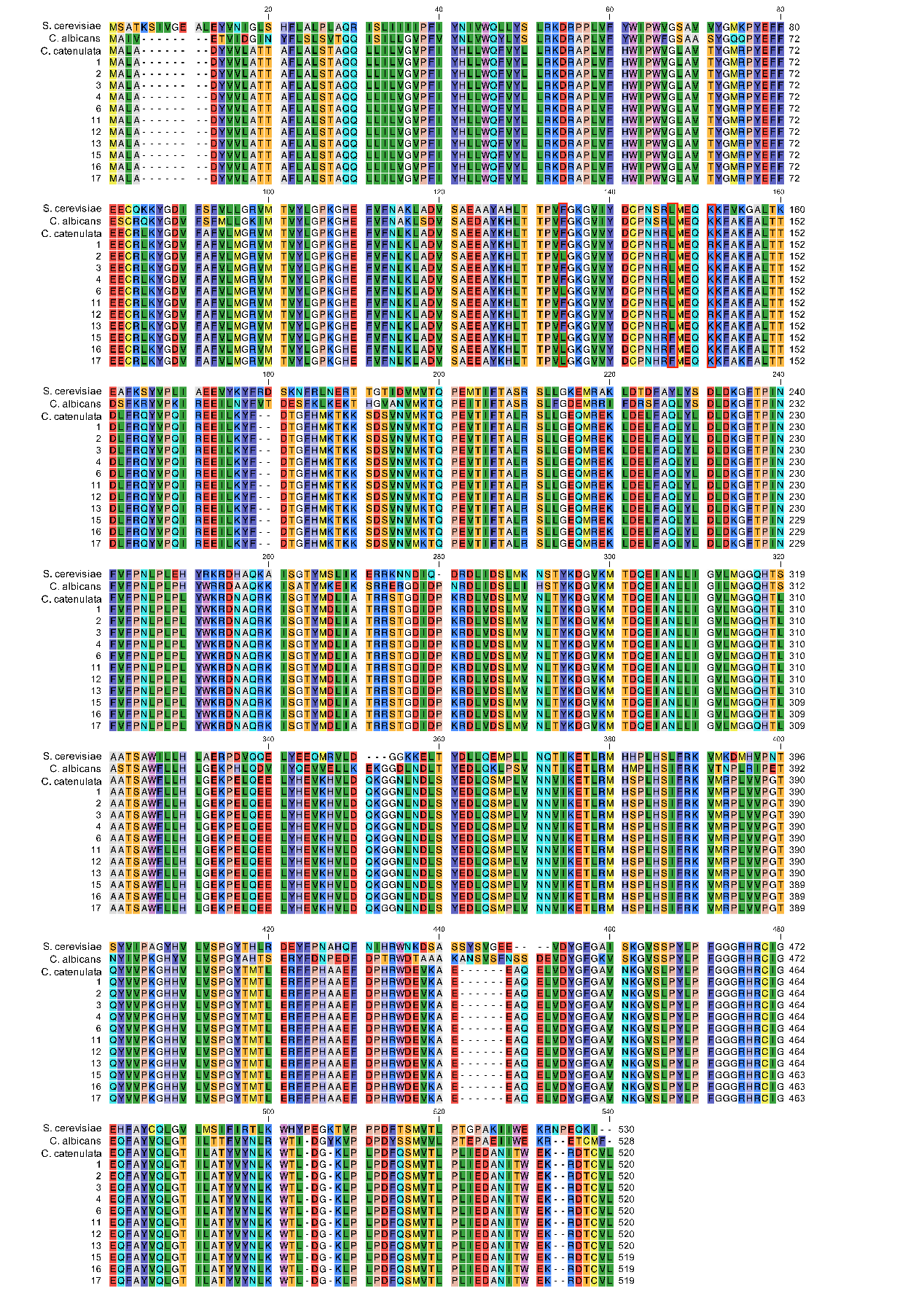


**Supplementary Figure 2.** Amino acid sequence alignment of *FKS1* from *Saccharomyces cerevisiae*, *Candida albicans*, and *Candida catenulata*. Three amino acid residues whose alterations had been linked to echinocandin resistance in *C. catenulata* are circled with red ovals.


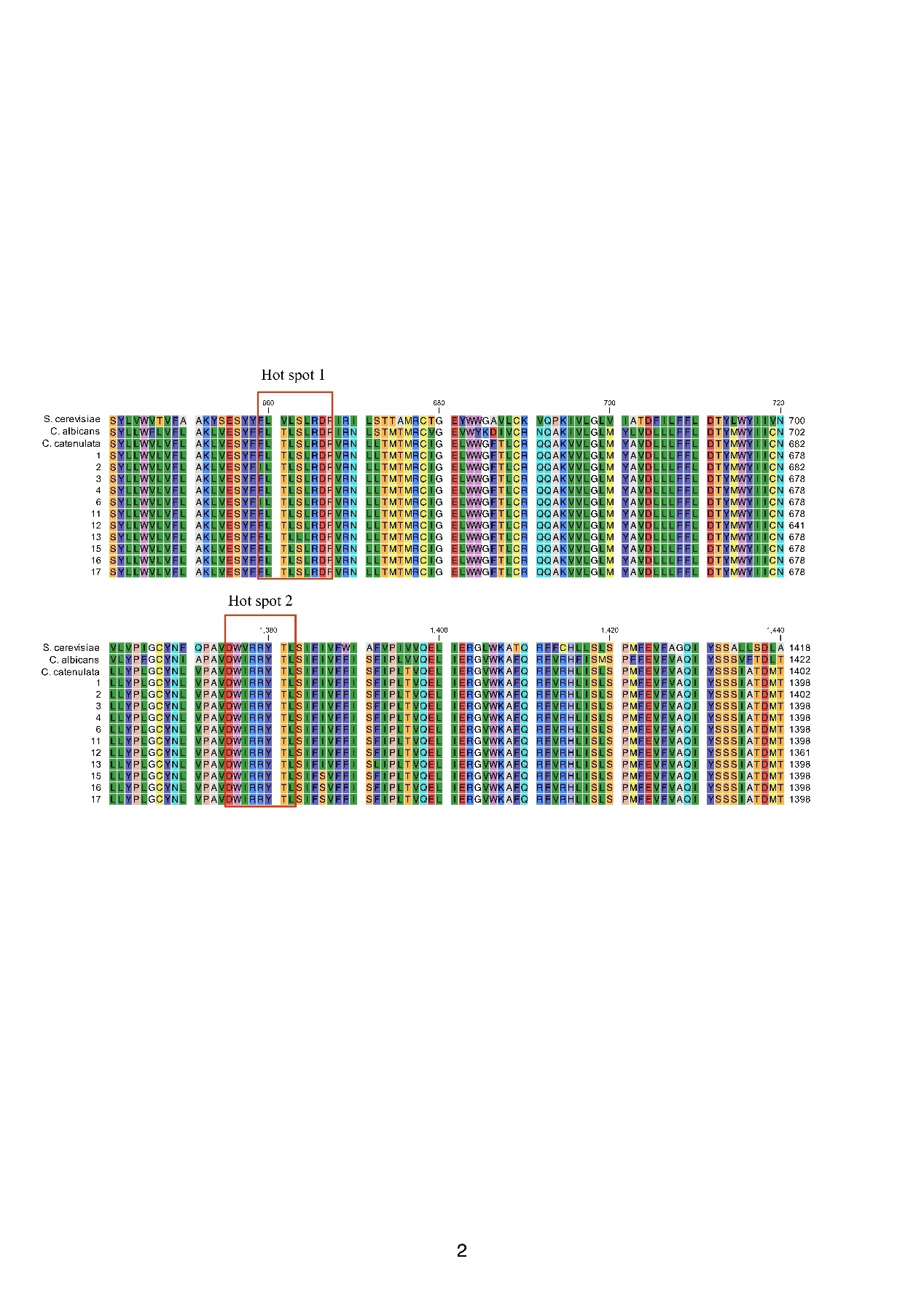


## Supplementary Tables

**Supplementary Table 1.** The forward primers and reverse primers for *FKS1*

| Number | Orientation | Sequence（5ʹ-3ʹ） |
| --- | --- | --- |
| 1 | F | GCACCCGATTTTCTACCTCA |
| 1 | R | GGTAAAGAGGAGTGATGACCC |
| 2 | F | GGAAGCTGAAGAAGGCTCGT |
| 2 | R | CTTACGCAGCGACTTGTTCAT |
| 3 | F | CCCAGAACTATGTGCAGACCA |
| 3 | R | CACGGGCAAAGGCTCCAAGAT |
| 4 | F | CGATTGGTCGCTCGTTCTAC |
| 4 | R | GCAAGGAAGCCCAAATACGAG |
| 5 | F | CTTTTGGAGTACTTGAAGCAG |
| 5 | R | TTAATCTTACCACCACGCAAC |
| 6 | F | TGCTGCTGGTAAGGAACAGAC |
| 6 | R | AAGAAGTCCTCCCAAGCAAAC |
| 7 | F | TCGTTGTCGCCCATGTTTGA |
| 7 | R | TCTTGAACTCACGGGTAAGA |
| 8 | F | TGTTGCGCTGGTCCCATGTT |
| 8 | R | CCCGGTCATCAACTCGTG |

**Supplementary Table 2.** Comparison of the MIC values obtained using the CLSI standard broth microdilution method and YeastOne™

| NO. | methods | MICs of antifungal drugs | | | | | | | | |
| --- | --- | --- | --- | --- | --- | --- | --- | --- | --- | --- |
|  |  | AND *^a^* | MF *^a^* | CAS *^a^* | FC *^a^* | PZ *^a^* | VOR *^a^* | IZ *^a^* | FZ *^a^* | AB *^a^* |
| 1 | YeastOne | 0.06 | 0.03 | 0.06 | <0.06 | 0.12 | 0.5 | 0.12 | 256 | 1 |
|  | CLSI | ND *^b^* | 0.12 | 1 | <0.06 | ND | 0.5 | ND | 128 | 0.25 |
| 2 | YeastOne | 1 | 0.5 | >8 | <0.06 | 0.12 | 1 | 0.12 | 64 | 1 |
|  | CLSI | ND | 2 | >8 | <0.06 | ND | 0.5 | ND | 64 | 1 |
| 3 | YeastOne | 0.25 | 0.12 | 4 | <0.06 | 0.06 | 0.12 | 0.12 | 4 | 0.5 |
|  | CLSI | ND | 0.25 | 2 | <0.06 | ND | 0.06 | ND | 2 | 0.5 |
| 4 | YeastOne | 0.06 | 0.015 | 0.12 | <0.06 | 0.06 | 0.12 | 0.06 | 4 | 1 |
|  | CLSI | ND | 0.06 | 0.5 | <0.06 | ND | 0.06 | ND | 4 | 0.5 |
| 6 | YeastOne | 1 | 0.12 | >8 | <0.06 | 0.06 | 0.25 | 0.06 | 32 | 1 |
|  | CLSI | ND | 1 | >8 | <0.06 | ND | 0.25 | ND | 32 | 1 |
| 11 | YeastOne | 0.12 | 0.03 | 0.5 | 0.12 | 0.06 | 0.12 | 0.06 | 4 | 1 |
|  | CLSI | ND | 0.12 | 1 | 0.12 | ND | 0.06 | ND | 2 | 1 |
| 12 | YeastOne | 0.12 | 0.03 | 4 | <0.06 | 0.12 | 0.25 | 0.12 | 64 | 0.5 |
|  | CLSI | ND | 0.12 | >8 | 0.12 | ND | 0.5 | ND | 64 | 0.5 |
| 13 | YeastOne | >8 | 8 | >8 | <0.06 | 0.03 | 0.06 | 0.06 | 4 | 0.5 |
|  | CLSI | ND | 8 | >8 | <0.06 | ND | 0.06 | ND | 2 | 0.5 |
| 15 | YeastOne | >8 | >8 | >8 | <0.06 | 0.12 | 4 | 0.12 | 256 | 0.5 |
|  | CLSI | ND | >8 | >8 | <0.06 | ND | 4 | ND | >256 | 0.5 |
| 16 | YeastOne | 4 | >8 | >8 | <0.06 | 0.06 | 1 | 0.12 | 128 | 0.5 |
|  | CLSI | ND | >8 | >8 | <0.06 | ND | 2 | ND | 128 | 0.5 |
| 17 | YeastOne | 4 | >8 | >8 | <0.06 | 0.06 | 0.5 | 0.06 | 64 | 0.25 |
|  | CLSI | ND | 2 | >8 | <0.06 | ND | 1 | ND | 64 | 0.5 |

*^a^* AND: Anidulafungin; MF: Micafungin; CAS: Caspofungin; FC: 5-Flucytosine; PZ: Posaconazole; VOR: Voriconazole; IZ: Itraconazole FZ: Fluconazole; AB: amphotericin B

*^b^* ND: no data

**Supplementary Table 3.** The variability of the *FKS1* gene in clinical *Diutina catenulata* isolates

| Isolate | Missense mutation | Synonymous mutation | MICs (μg/mL) | | |
| --- | --- | --- | --- | --- | --- |
|  | Nucleotide mutation (amino acid mutation) |  | AND *^a^* | MF *^a^* | CAS *^a^* |
| 1 | G3367A(G1123S), C5006T(A1669V) | A591G, G732C, T1119C, C1194T, G1203A, G1365T, A1554G, T1605C, T2079C, C2100T, C2682T, C2781G, G2871A | 0.06 | 0.03 | 0.06 |
| 2 | T1861A(F621I), G3367A(G1123S) | A591G, G732C, T1119C, C1194T, G1203A, G1365T, A1554G, T1605C, T1861A, T2079C, C2100T, C2682T, C2781G, G2871A | 1 | 0.5 | >8 |
| 3 |  | A591G, G732C, T1119C, G1365T, T1605C, T2079C, C2682T, G3036A, G4230C, C4704T, T4977C, T5088C | 0.25 | 0.12 | 4 |
| 4 | G3367A(G1123S) | A591G, G732C, T1119C, C1194T, G1203A, G1365T, A1554G, T1605C, T2079C, C2100T, C2682T, C2781G, G2871A | 0.06 | 0.015 | 0.12 |
| 6 | T1861A(F621I), G3367A(G1123S) | A591G, G732C, T1119C, C1194T, G1203A, G1365T, A1554G, T1605C, T2079C, C2100T, C2682T, C2781G, G2871A | 1 | 0.12 | >8 |
| 11 | C4462T(R1488C) |  | 0.12 | 0.03 | 0.5 |
| 12 | G3367A(G1123S) | A591G, G732C, T1119C, C1194T, G1203A, C1299T, G1365T, A1554G, T1605C, T2079C, C2100T, C2682T, C2781G, G2871A, C3273T | 0.12 | 0.03 | 4 |
| 13 | C1874T(S625L), T4062G(F1354L) | A591G, G732C, T1119C, G1365T, T1605C, T2079C, C2682T, G3036A, G4230C, C4704T, T4977C, T5088C | >8 | 8 | >8 |
| 15 | G3367A(G1123S), T4043G(I1348S) | A591G, G732C, T1119C, C1194T, G1203A, G1365T, A1554G, T1605C, T2079C, C2100T, C2682T, C2781G, G2871A | >8 | >8 | >8 |
| 16 | G3367A(G1123S), T4043G(I1349S) | A591G, G732C, T1119C, C1194T, G1203A, G1365T, A1554G, T1605C, T2079C, C2100T, C2682T, C2781G, G2871A | 4 | >8 | >8 |
| 17 | G3367A(G1123S), T4043G(I1349S) | A591G, G732C, T1119C, C1194T, G1203A, G1365T, A1554G, T1605C, T2079C, C2100T, C2682T, C2781G, G2871A | 4 | >8 | >8 |

*^a^* AND: anidulafungin; MF: micafungin; CAS: caspofungin

**Supplementary Table 4.** Clinical information obtained from literature review of *Diutina catenulata*

|  | | | | | | | | | |
| --- | --- | --- | --- | --- | --- | --- | --- | --- | --- |
| No. | Publication year | Location | Age/Sex | Specimen type | Type of infection | Predisposing factors | Treatment option | Outcome | reference |
| 1 | 1977 | [Australia](http://www.baidu.com/link?url=5Kj1c0PmU3BqTrY2raLXVYLXxKyU2FYVgzqKbiePLGBpPZbZyH14SIkHBr726j3zPD129Iv_-HzihfQ0X17B11IJSIfw-FuiX13979YulE3&wd=&eqid=a64c11b1001441ca000000036138d2ca) | 50 years/Male | Clippings and scrapings | onychomycosis | ND a | ND a | Survived | Crozier, 1977; |
| 2 | 1999 | France | 42 years/Female | Blood | Candidemia | gastric carcinoma, immunosuppressive therapy, and broad-spectrum antibiotic therapy | ﬂuconazole ，Amphotericin B, | Died | Radosavljevic et al., 1999 |
| 3 | 2018 | USA | 37 years/Male | Blood | Candidemia | broad-spectrum antibiotic therapy | fluconazole，micafungin | Survived | Ha et al., 2018 |

*^a^* ND: no data
